# Advances in Supportive Care for Multiple Myeloma-Related Bone Disease—A Review

**DOI:** 10.3390/cancers17132166

**Published:** 2025-06-27

**Authors:** Saadia Abbas, Haleema Sadia Adil, Shahzad Raza, Janita Basit, Karan Arul, Faiz Anwer, Muhammad Hamza Habib

**Affiliations:** 1 Medical College, Aga Khan University, Karachi 74800, Pakistan; 2 University College London Medical School, London WC1E 6DE, UK; 3 Department of Hematology-Oncology, Cleveland Clinic, Taussig Cancer Institute, Cleveland, OH 44106, USA; 4 Ohio State University Wexner Medical Center, Columbus, OH 43210, USA; 5 Rutgers Cancer Institute of New Jersey, Robert Wood Johnson Medical School, New Brunswick, NJ 08901, USA

**Keywords:** supportive care, myeloma bone disease, bone pain, myeloma bone pain, palliative care

## Abstract

Multiple myeloma (MM) is an incurable plasma cell malignancy characterized by the increased production of monoclonal immunoglobulin. Patients with MM are at high risk of suffering from disease-related complications. Osteolytic bone disease is one of the most common disease-related complications, resulting in chronic pain and skeletal pathologies that contribute significantly to high morbidity and mortality rates among MM patients. In addition to standard anti-MM therapy, management of disease-related sequelae is integral to improving quality of life in MM patients. Bisphosphates have long been the mainstay treatment for patients with myeloma bone disease (MBD) due to their ability to reduce the incidence of skeletal-related adverse events. However, in recent years, a deeper understanding of the complex biology and pathophysiology associated with myeloma bone disease has led to the development of novel therapies that have the potential to improve MM patient management and outcomes. This narrative review uses the most recent extant publications to review all such advancements. It aims to summarize evidence-based strategies for the management of myeloma bone disease and therapy-associated adverse events whilst highlighting current guidelines on optimal bisphosphonate use and providing an overview of promising new agents currently in clinical development.

## Simple Summary

Multiple myeloma is a blood cancer of plasma cells that can cause significant kidney and nerve damage, and crucially, bone damage in 85% of patients. This can cause pain, fractures, and affect the quality of life of patients. Whilst managing cancer is important, it is also crucial to manage the disease’s complications, specifically the bone disease. Currently, regimens focus on protecting bones with bisphosphonate medications, pain relief using a combination of analgesia, radiotherapy, and surgical procedures that can protect the bones. As our understanding of the pathophysiology behind the bone disease has developed, trials are investigating new treatment options, such as a variety of monoclonal antibodies, to aid in bone formation whilst improving pain relief for these patients. This work examines current treatments and novel approaches to guide healthcare professionals in the management of bone diseases observed in patients with myeloma.

## 1. Introduction

Multiple myeloma (MM) is the second most common hematologic malignancy after non-Hodgkin lymphoma, with an annual incidence of approximately 3–4 cases per 100,000 individuals in the United States. The disease predominantly affects older adults, with a median age at diagnosis of 65 years, and demonstrates a slight male predominance. MM ranks as the 14th most common cause of cancer deaths in the United States [1].

Pathologically, MM is defined by the clonal proliferation of malignant plasma cells within the bone marrow and the overproduction of monoclonal immunoglobulins (paraproteins), culminating in a spectrum of end-organ damage. The classical presentation is encapsulated by the “CRAB” criteria: hypercalcemia, renal impairment, anemia, and osteolytic bone lesions. Among these, myeloma bone disease (MBD) is both prevalent and clinically significant, contributing substantially to morbidity and diminished quality of life.

Chronic pain is a pervasive and debilitating symptom, affecting up to 73% of patients across all stages of MM [2]. Pain may arise from multiple etiologies, including MBD (in the form of osteolytic lesions or pathologic fractures); chemotherapy-induced peripheral neuropathy (CIPN); immunosuppression-related complications (such as herpes zoster reactivation); and long-term survivorship issues (i.e., cancer survivor syndrome characterized by cognitive, physical, and psychological sequelae) [1,2]. Amongst these, MBD is the most frequent and severe contributor to disease burden, closely associated with reduced functional capacity, impaired quality of life, and decreased overall survival (OS) [3].

Without effective therapy, the median OS following diagnosis is approximately 50 months, with symptomatic patients experiencing a median survival of less than six months [4]. Consequently, all patients diagnosed with MM will require treatment. The primary goals of MM management are twofold: addressing disease progression and managing disease-related sequelae [5]. Achieving these objectives requires a multidisciplinary approach encompassing hematology/oncology, radiation oncology, surgical intervention, pain and palliative care, and rehabilitation services with a combination of MM-directed treatment and supportive therapies.

This narrative review provides a comprehensive synthesis of the pathophysiology and management of chronic pain and therapy-related adverse events associated with MBD. This approach has enabled the integration of evidence from a wide range of disciplines and clinical perspectives. We also discuss palliative and supportive care strategies that can be adopted when caring for MM patients with MBD. Overall, this review aims to provide an overview for pain and non-pain specialists to address chronic pain among this complex patient population.

## 2. Methods and Materials

### 2.1. Search Strategy and Selection Criteria

A comprehensive literature review was undertaken to synthesize current evidence on the supportive care and management of myeloma bone disease (MBD) in patients with multiple myeloma. Searches were conducted through PubMed focusing on studies published in English from 2000 to 2024.

The search strategy employed the following free-text keywords: “Multiple Myeloma”, “Myeloma Bone Disease”, “Bone Pain”, “Osteolytic Lesions”, and “Skeletal-Related Events”.

An initial yield of approximately 780 articles (including clinical trials, randomized clinical trials, meta-analyses, observational studies, systematic reviews, scoping reviews) was retrieved. The quality of these papers was reviewed and ensured that relevant guidelines were used for the respective study types, including PRISMA, CONSORT, and STROBE. Further screening of titles, abstracts, and full-text articles was evaluated for relevance, contribution to clinical understanding, clinical manifestations, and management strategies with a focus specifically on MBD. A total of 86 studies were included in the final synthesis.

#### 2.1.1. Level of Evidence

The included literature encompassed a range of study designs, classified according to the Oxford Centre for Evidence-Based Medicine Levels of Evidence. The majority consisted of Level 2 and Level 3 studies, including randomized controlled trials, prospective and retrospective cohort studies, and expert consensus guidelines. A smaller proportion comprised Level 1 evidence (systematic reviews and meta-analyses) and Level 4 evidence (case series and expert opinion).

#### 2.1.2. Study Design and Review Approach

This study constitutes a narrative review of the literature, synthesizing available evidence to highlight the current standards, therapeutic options, and emerging novel treatments for bone disease in MM. No formal meta-analytic techniques were applied. Instead, qualitative analysis was undertaken to evaluate clinical trends, identify best practices, and highlight ongoing gaps in evidence that warrant future investigation.

### 2.2. Myeloma Bone Disease (MBD)

Osteolytic bone disease affects up to 80% of patients with newly diagnosed MM, presenting with disability, morbidity, and an increased risk of skeletal-related events (SRE), such as pathological fractures and spinal cord compression [3]. MBD is a significant contributor to the disease burden associated with MM. Therefore, concentrated efforts should be made to manage and prevent MBD.

For newly diagnosed or suspected MM patients, imaging is crucial for the identification and assessment of MBD. These images typically reveal discrete lytic lesions (i.e., plasmacytomas) that involve the skull, vertebrae, or long bones and diffuse osteopenia [3].

According to recommendations by the International Myeloma Working Group (IMWG), the first-choice imaging tool for the diagnosis of disease-related bone lesions is whole-body low-dose CT. Notably, conventional plain radiographs are no longer recommended for diagnosis, as they fail to detect up to 25% of lytic lesions due to low sensitivity. However, PET-CT may be used as an alternative diagnostic imaging modality if whole-body low-dose CT scanning is not available. If whole-body CT is negative and other myeloma-defining events are absent, whole-body MRI (or MRI of the spine and the pelvis, if whole-body MRI is not available) is recommended as the next diagnostic step because of its high sensitivity and the necessity to exclude focal lesions as myeloma-defining events [6]. For the follow-up of disease progression and the monitoring of treatment response, PET-CT is the gold standard. This functional imaging can detect metabolically active lesions earlier than changes seen on MRI or CT scans, particularly in the context of minimal residual disease (MRD) and non-secretory or oligosecretory disease, as a non-invasive technique [7]. Moreover, this imaging can differentiate between active residual disease and inactive sclerotic lesions that can persist following treatment. Additionally, a complete metabolic response on PET/CT is associated with significantly improved OS and PFS. Several studies have reported a sensitivity and specificity for the detection of bone lesions in the range of 80% to 100% for PET-CT [8]. According to several comparative studies, MRI and PET-CT show a similar sensitivity for the detection of focal bone marrow involvement [9].

### 2.3. Pathophysiology of Bone Disease in Multiple Myeloma

The disease process involves an interplay between MM cells and three main cell types: osteoblasts, osteoclasts, and osteocytes [10,11].

Normal physiological bone remodeling is characterized by the action of osteoblasts, which differentiate from mesenchymal stem cells in the periosteum. This differentiation of osteoblasts depends on transcription factors such as Runx2/Cbfa1 and osterix. Conversely, osteoclasts are large, multinucleated cells derived from the monocyte and macrophage lineage, responsible for the proteolytic degradation and decalcification of bone [11,12]. The balance between these two cell types, along with structural regulation, depends on the activity of interleukins, tumor necrosis factors (TNF), colony-stimulating factors, and growth factors in the marrow microenvironment [12]. Under normal physiological conditions, the differentiation, activation, and maturation of osteoclasts is prompted by binding of the RANK receptor to the nuclear factor kappa-B ligand (RANKL), a cytokine and TNF family protein expressed by osteoblasts. Osteoblastic expression of TNF family proteins can also downregulate osteoclast activity, particularly through osteoprotegerin (OPG) expression. OPG serves as a decoy that prevents interaction between RANK and RANKL, which can minimize osteoclast-mediated bone degradation [12,13].

In the MM disease state, this coupling mechanism of osteoclasts and osteoblasts is lost due to a combination of increased osteoclastogenesis and suppressed osteoblastic activity, owing to the osteoclast-activating and osteoblast-inhibiting factors produced by the myeloma plasma cells, respectively. Myeloma plasma cells secrete the Wnt signaling pathway inhibitor Dickkopf1 (DKK1), which directly inhibits osteoblast differentiation. Secreted Frizzled-related proteins (sFRPs) also negatively regulate the Wnt signaling pathway and have been implicated in the suppression of osteoblast differentiation, which causes uncoupled bone remodeling and, consequently, bone resorption [14]. IL-7, secreted by bone marrow stromal cells, suppresses the Runx2/Cbfa1 promoter activity, which is a transcription factor for osteoblast formation. The suppression of Runx2/Cbfa1 results in unregulated osteoclast differentiation and expansion [13]. The tumor plasma cells secrete humoral factors, including IL-1, IL-3, prostaglandins, and TNF-α, which upregulate RANKL expression, causing increased bone resorption. These imbalances in bone metabolism have important implications clinically, resulting in chronic pain, acute or subacute fractures, lytic lesions, and hypercalcemia of malignancy [15].

#### Serum Markers in Myeloma Bone Disease

Continuous bone remodeling generates biochemical markers that reflect bone resorption and formation activity. In the MM disease state, increased osteoclast activity compared to osteoblast activity leads to an imbalance in their serum levels [16].

Bone resorption markers are derived from type I collagen degradation and include urinary hydroxyproline, hydroxylysine glycosides, pyridinoline (PYD), deoxypyridinoline (DPD) crosslinks of type I collagen, amino- and carboxy-terminal peptide fragments (NTX and CTX, respectively), as well as C-terminal telopeptide generated by matrix metalloproteinases (ICTP) [17]. The α-aspartic acid present in the C-terminal telopeptides is further converted to the β form (β-CTx), which is a specific and stable marker of bone resorption, indicating initial stages of type I collagen degradation [16]. Novel markers, such as serum tartrate-resistant acid phosphatase isoform type 5b (TRACP-5b) and bone sialoprotein (BSP), a non-collagenous extracellular protein of mineralized tissues, have also been shown to reflect bone resorption processes [18].

On the other hand, bone formation markers that enter circulation, reflecting osteoblast activity, include serum bone-specific alkaline phosphatase (ALP), osteocalcin (OC), and type I collagen propeptides, such as procollagen type I N-propeptide (PINP) and C-propeptide (PICP) [17].

These serum markers offer an inexpensive method for evaluating the extent of bone disease in MM, assessing treatment response, and estimating the risk of skeletal complications in MM patients. Studies have demonstrated elevated levels of bone resorption markers, including urinary PYD, DPD, NTX, and serum levels of CTX, ICTP, and TRACP-5b in MM patients compared to healthy controls. These levels have been correlated with the severity of osteolytic disease [17]. Among these, serum ICTP and urinary NTX have been found to most accurately reflect the extent and progression of MBD [19]. A randomized phase III study with 282 MM patients revealed a significant association between urinary NTX levels and the risk of developing the first SRE, with a 68% increase in risk for every 100-unit increase in NTX [20]. Additionally, studies have shown higher levels of serum ICTP and urinary NTX in advanced stages of MM compared to early-stage disease, making these markers important for prognosis [21].

Although bone formation markers have shown variable prognostic value when used alone, recent studies have suggested they can support therapeutic monitoring when combined with bone resorption markers for risk stratification. The resorption markers are better established in correlating with both disease activity and the risk of SREs. The IMWG has acknowledged its role in emerging frameworks for bone-targeted therapies [22,23].

### 2.4. Management of Myeloma Bone Disease

#### 2.4.1. Antiresorptive Therapy

Antiresorptive therapies, namely bisphosphonates and denosumab, represent the gold standard for the prevention and management of skeletal complications in MM. Table 1 highlights the different antiresorptives being used for MBD and novel therapies being explored.

#### 2.4.2. Bisphosphonates

Bisphosphonates are pyrophosphate analogs that bind to exposed areas of hydroxyapatite crystals during the bone remodeling process. They act as potent inhibitors of bone breakdown by incorporating into the bone matrix and inducing osteoclast apoptosis, which prevents their differentiation and expansion [24]. The American Society of Clinical Oncology (ASCO), International Myeloma Working Group (IMWG), European Myeloma Network (EMN), and National Comprehensive Cancer Network (NCCN) recommend bisphosphonate administration to all patients with active MM, regardless of the presence or absence of MBD [25,26,27]. This recommendation is supported by evidence from an updated meta-analysis of 7293 participants across 24 randomized controlled trials. Twenty of these trials compared bisphosphonates to a placebo or no treatment, while the remaining four compared different bisphosphonates. The meta-analysis demonstrated the favorable effects of bisphosphonates in preventing SREs (relative risk [RR] 0.74, 95% confidence interval [CI] 0.63–0.88), preventing pathological vertebral fractures (RR 0.74, 95% CI 0.62–0.89), and reducing bone pain indices (RR 0.75, 95% CI 0.60–0.95) [28].

Zoledronic acid (ZA) and pamidronate are the two main bisphosphonates approved by the FDA for preventing osteolytic lesions in MM. Pamidronate is typically administered intravenously at 90 mg every 4 weeks. At this dose, MM patients with at least one osteolytic lesion see a significant reduction in SREs from 41% to 24% (*p* < 0.001). Additionally, MM patients report reduced pain and increased quality of life after the first month of pamidronate use [29]. While pamidronate yields a clear therapeutic benefit for bone disease, there are several therapy-related adverse effects associated with its use. Therefore, dosing strategies that minimize adverse side effects without compromising therapeutic benefits are important. A randomized double-blinded study of 504 patients compared 30 mg of pamidronate administered over 45 min with the traditional standard dose of 90 mg. Whilst both treatment groups had similar SRE rates, the 30 mg group reported fewer therapy-related adverse events, such as jaw osteonecrosis and nephrotoxicity [30].

ZA, a more potent bisphosphonate, is typically administered intravenously at 4 mg every 4 weeks with a shorter infusion time (15 versus 45 min) and, therefore, may be more convenient [31]. ZA has demonstrated efficacy in preventing SREs and is the only bisphosphonate to show an OS advantage, which exceeds both pamidronate and clodronic acid [29,32].

Additionally, its potent antiresorptive activity and anti-MM effect make this the preferred agent in clinical settings. Particularly, ZA has shown both progression-free survival (PFS) and OS benefit compared to placebo or no treatment. ZA administration showed a PFS benefit (hazard ratio [HR] 0.70 [95% CI 0.52–0.95]) and OS benefit (HR 0.57 [95% CI 0.43–0.75]) compared to a control in Mhaskar’s meta-analysis [28]. The randomized Medical Research Council (MRC) Myeloma IX trial compared ZA administered to 981 patients with oral clodronate administered at 1600 mg daily to 979 patients. This trial concluded that ZA reduced mortality by 16% (HR 0.84, 95% CI 0.74–0.96; *p* = 0.012), improved PFS by 12% (HR 0.88, 95% CI 0.80–0.98; *p* = 0.018), increased median OS from 44.5 months to 50.0 months (*p* = 0.04), and resulted in a lower SRE incidence (27% vs. 35%; *p* = 0.0004) [32]. While skeletal morbidity improved in MM patients with and without preexisting bone lesions, survival benefits were only seen in patients with pre-existing lesions. The mechanism behind ZA anti-MM activity is attributed to the inhibition of protein prenylation and reduced expression of bone marrow stromal cell-associated adhesion molecules, thereby inducing apoptosis in myeloma cells [33].

Bisphosphonates are also indicated for the treatment of myeloma-related hypercalcemia of malignancy, whereby zoledronate is regarded as superior to pamidronate [34]. With coexisting osteoporosis, bisphosphonates can be considered to address microarchitectural bone changes that arise in the initial stages of myelomagenesis, such as in smoldering multiple myeloma (SMM), monoclonal gammopathy of undetermined significance (MGUS), or solitary plasmacytoma [35]. In patients with MGUS and osteoporosis, intravenous administration of ZA every 6 months and weekly oral administration of alendronic acid were shown to increase bone mineral density [36]. Additionally, in patients with SMM, monthly IV treatment with ZA or pamidronate for a minimum of one year was shown to significantly reduce SRE incidence at the time of progression to symptomatic multiple myeloma [37].

#### 2.4.3. Duration of Treatment

The introduction of novel therapies, such as quadruplet combinations (i.e., anti-CD38 monoclonal antibodies), produces deeper and more durable disease remissions, questioning the necessity of prolonged bisphosphonate treatment beyond one year [38]. The Z-MARK trial investigated a biomarker-directed strategy with ZA dosing every 3 months in patients with less than 50 nmol/mmol urinary N-telopeptide levels, which demonstrated a reduced incidence of SREs and ONJ [39]. Moreover, the OPTIMIZE-2 trial demonstrated that after at least 12 months of monthly treatment, ZA administration every 12 weeks maintained efficacy in preventing SREs and was associated with fewer renal and bone-related toxicities, supporting individualized, biomarker-informed de-escalation strategies [40]. For the majority of MM patients, the guidance recommends monthly dosing with ZA alongside monitoring of bone turnover markers.

Furthermore, the MRC Myeloma IX trial demonstrated that patients who received zoledronic acid for two years or more had improved OS compared to those receiving clodronic acid [32]. They also found that continuation of ZA therapy beyond two years did not grant any greater benefit in OS [36]. Moreover, the MAGNOLIA randomized trial demonstrated that continuing monthly ZA beyond two years significantly reduced the risk of SREs over years 3 and 4 (hazard ratio 0.40), with no observed difference in OS or substantially increased toxicity, thus supporting extended treatment duration in appropriately selected MM patients [41]. Given no improved benefit in OS, and the potential for adverse effects with prolonged therapy, dosing frequency should be individualized and continuously re-evaluated, taking into consideration fracture risk, patient demographics, bone mineral density, risk of osteonecrosis of the jaw (ONJ), and the presence of secondary osteoporosis. Current IMWG guidelines recommend monthly ZA administration for at least 12 months in MM patients. Based on osteoporosis guidelines, if the patient achieves a very good partial response (VGPR) or better at 12 months, the physician should consider reducing the dosing frequency from monthly to every three to six months or discontinuing ZA altogether. If a VGPR is not achieved within 12 months, monthly administration of ZA should continue until this response level is reached, after which time the dosing frequency can be reduced [42].

Similarly, treatment duration for pamidronate in patients with active disease should be continuously monitored and re-evaluated, considering patient and disease-related risk factors to reduce dosing intensity without compromising efficacy [38]. In cases of biochemical relapse, zoledronic or pamidronic acid should be reinitiated to mitigate the risk of new bone events at clinical relapse. In short, the duration and frequency of bisphosphonate therapy for bone disease in MM should be tailored to patient response and risk factors.

#### 2.4.4. Denosumab

Denosumab is a human monoclonal IgG2 antibody that targets RANKL with high affinity and inhibits the RANKL-RANK interaction crucial for osteoclastogenesis and bone resorption [43]. Inhibition of the RANKL-RANK interaction decreases bone resorption, increases cortical and cancellous bone mass, and improves trabecular microarchitecture [44]. According to Raje et al., denosumab may have anti-MM activity, inhibiting myeloma growth and reactivation of dormant MM cells [45].

The efficacy of denosumab in MM has been validated in multiple trials. In a Phase I trial, denosumab demonstrated a sustained reduction in bone resorption markers compared to intravenous pamidronate [46]. Subsequent Phase II and III trials further established the ability of denosumab to effectively inhibit RANKL, suppress bone turnover, and reduce the incidence of SREs, even in patients refractory to BP therapy [47]. The NCT01345019 Phase III trial, involving 1718 newly diagnosed MM patients, demonstrated the non-inferiority of denosumab compared to ZA in delaying the first SRE (HR 0·98 [95% CI 0·85–1·14]; *p* = 0·010) after a median duration of 17·3 months (IQR 8·9–28∙5) for denosumab and 17·6 months (9∙4–28∙1) for ZA. Both treatment groups had comparable OS and reduced incidence of renal-related adverse events [48].

Denosumab is currently approved and recommended for the treatment of newly diagnosed MM, and for patients with relapsed or refractory MM with evidence of multiple myeloma-related bone disease. The current dosing recommends monthly 120 mg subcutaneous injections to ensure RANKL inhibition and skeletal protection. In patients with smoldering MM or plasmacytomas and concurrent osteoporosis, 60 mg subcutaneously is recommended in accordance with the osteoporosis guidelines [42].

#### 2.4.5. Duration of Treatment

Denosumab is recommended for continuous administration until unacceptable toxicity occurs. Notably, discontinuation can only be considered after 24 months of treatment if the patient achieves a VGPR or better with anti-myeloma treatment. The decision to discontinue denosumab should be based on patient characteristics. The discontinuation of denosumab can also have a rebound effect as it does not bind to bone, unlike bisphosphonates, making its effects reversible. This results in increased osteoclastogenesis due to increased RANKL activity with an elevation in bone resorption markers, including CTX, with a rapid loss of BMD. Consequently, there is an increased risk of vertebral fractures seen within 12–24 months following discontinuation [49]. This rebound effect with discontinuation has been well documented in osteoporosis patients and should be accounted for when considering discontinuation in MM patients. To address this concern, a single dose of IV bisphosphonates, such as ZA, can be administered after at least 6 months. Alternatively, denosumab could be administered every six months as a preventive measure [42,50].

### 2.5. Choice of Antiresorptive Therapy

#### 2.5.1. In Patients with Renal Impairment

Renal impairment is a serious complication in MM, affecting 20–40% of newly diagnosed patients [51,52]. The most common cause of renal dysfunction in MM patients is cast nephropathy, secondary to excessive light chain production [53]. Additionally, hypercalcemia, dehydration, the use of nephrotoxic drugs (such as aminoglycoside antibiotics, nonsteroidal anti-inflammatory agents, and radiocontrast), and monoclonal immunoglobulin-related glomerulopathies further potentiate the insult and injury caused by light chains [54].

Bisphosphonates, the cornerstone treatment for bone disease in MM, are associated with potential renal toxicity, making their use in patients with pre-existing renal dysfunction challenging. ZA has been implicated in the development of both acute and chronic kidney injury, the most common underlying pathology being acute tubular necrosis [55]. Renal toxicity with ZA is dose and infusion-time-dependent, as rapid infusion or higher doses increase intracellular ZA concentration, leading to higher toxicity [56]. Pamidronate therapy also poses challenges for renal function and can result in several renal conditions, including collapsing focal segmental glomerulosclerosis (CFSGS) and tubulointerstitial nephritis [57]. Hence, when using bisphosphonates, careful monitoring, dose adjustments, and appropriate management strategies are essential to minimize the risk of renal complications. Particularly, in patients with creatinine clearance between 30 mL/min and 60 mL/min, dose adjustments for zoledronate and clodronic acid are necessary, and pamidronate should be administered over an extended duration of 4 h [42]. Zoledronate and pamidronate should only be administered when creatinine clearance exceeds 30 mL/min, while clodronic acid should only be used when creatinine clearance exceeds 12 mL/min. The reinitiation of bisphosphonate therapy should be considered when serum creatinine levels return to within 10% of baseline values [42].

Unlike bisphosphonates, denosumab does not require renal clearance and is not associated with renal toxicity. Therefore, denosumab is a safer alternative for patients who already have compromised renal function and those at high risk of developing renal compromise due to disease or therapy-related complications [58]. Clinical trials, including the 20,090,482 study, have demonstrated that adverse events related to renal function among patients with a creatinine clearance between 30 and 60 mL/min were doubled with ZA (26%) compared to denosumab (13%) [47]. Therefore, in patients at elevated risk for renal toxicity or those who develop renal impairment during bisphosphonate therapy, switching to denosumab is strongly recommended [42].

#### 2.5.2. In Patients with Hypercalcemia

Hypercalcemia occurs in up to 10% of MM patients [59]. Severe hypercalcemia is a metabolic emergency caused by increased osteoclastic bone resorption, with serum corrected calcium being greater than 3.5 mmol/L [59]. This requires urgent treatment to prevent neurological, cardiac, and renal complications, which includes IV hydration followed by IV bisphosphonates. For hypercalcemia of malignancy, bisphosphonates, particularly ZA, have long been established as the treatment of choice. In addition to their role in rehydration and disease control, bisphosphonates are also potent inhibitors of bone resorption, ZA being more effective than pamidronate and ibandronate in this regard [34,60]. For patients who are unresponsive to bisphosphonate therapy, denosumab is considered the second-line treatment. Denosumab is also the first-line treatment in patients with renal dysfunction, with continuous monitoring and evaluation of dosing frequency, due to an increased risk of hypocalcemia [61,62]. In patients with mild renal impairment, ZA may be administered cautiously with initial hydration, renal dosing adjustments (3–4 mg over 15–30 min, possibly at lower doses), and close monitoring, as supported by the IMWG recommendations [42]. However, if renal dysfunction is more advanced, alternatives such as denosumab or hydration and corticosteroids can be employed to reduce the risk of nephrotoxicity.

### 2.6. Monitoring and Management of Adverse Effects

#### 2.6.1. Serum Monitoring and Supplementation

Regular monitoring of serum electrolytes, including calcium, phosphate, magnesium, and vitamin D3 levels, is crucial for patients with MM receiving bone-modifying agents [43,47]. Baseline vitamin D levels should be assessed before initiating supplementation. If vitamin D levels are low < 30, then higher doses of vitamin D, such as 50,000 IU units weekly for 8 weeks, should be considered. If vitamin D levels are >30, then higher doses of 1000–2000 IU daily should be used for health maintenance [63]. In the NCT01345019 trial, hypocalcemia was seen more frequently with denosumab (17%) than zoledronic acid (12%) [48]. Daily supplementation with calcium (600 mg) and vitamin D3 is recommended to prevent severe hypocalcemia [64], particularly since vitamin D deficiency is common in MM patients [65]. Although previous guidelines recommended 400 IU daily, current evidence has highlighted 800–2000 IU daily [42]. In cases of chronic kidney disease, active forms such as calcitriol (0.25–1 mcg/day) or alfacalcidol (0.25–1 mcg/day) may be necessary to support mineral metabolism and mitigate skeletal complications, as these patients can have impaired activation of vitamin D. However, in cases of hypercalcemia, supplementation should only be initiated after the normalization of serum calcium levels with close monitoring of patients with renal impairment receiving calcium supplements [42].

#### 2.6.2. Renal Function Monitoring

Routine monthly evaluation of creatinine clearance, serum electrolytes, and urinary albumin (especially in patients receiving pamidronate due to its glomerular toxicity) is essential due to the risk of bisphosphonate-induced acute renal damage [66].

#### 2.6.3. Osteonecrosis of the Jaw (ONJ)

ONJ is traditionally defined as exposed, necrotic bone in the jaw that does not heal after 8 weeks and is often painful [67]. The risk of ONJ is higher following dental extractions and other invasive oral procedures [68]. To reduce the risk, a comprehensive dental examination and completion of any necessary invasive treatments should be performed before initiating bisphosphonates [69]. ONJ is a serious but uncommon complication associated with long-term use of antiresorptive medications. ZA has a 4.85-fold higher risk of ONJ compared to alendronate and pamidronate over 5 years, owing to its higher potency than other bisphosphonates [70]. ONJ rates with denosumab range from 1.8 to 4.3% and from 1.3 to 15% with ZA. However, analysis has shown no statistically significant difference between these two agents [48]. Previously, expert opinion suggested temporary discontinuation could reduce ONJ risk; however, Aboalela et al. found insufficient evidence that drug holidays reduce ONJ incidence in cancer patients on high-dose bisphosphonates or denosumab [71]. Moreover, a randomized clinical trial by Ottesen et al. showed no significant difference in ONJ occurrence between patients who continued versus paused antiresorptive therapy during dental procedures. Similarly, a 2023 MDPI review concluded that drug holidays do not significantly mitigate ONJ risk, cautioning against routine interruption of therapy. Both ESMO and IMWG guidelines currently advise that antiresorptive therapy should not be routinely interrupted for ONJ prevention; however, therapy may be temporarily withheld in confirmed cases until healing occurs [42]. This reflects a growing consensus to individualize decisions based on clinical context rather than applying routine drug discontinuation. In 2017, guidelines suggested bisphosphonates should be discontinued if ONJ occurs; however, treatment may be resumed after healing, especially in cases of progressive lytic bone disease or recurrent hypercalcemia [72]. For patients undergoing elective invasive dental procedures (e.g., tooth extractions, dental implants), the IMWG guidelines recommend stopping bisphosphonate therapy for 3 months before and after such procedures, while denosumab should be discontinued for 30 days before and reinitiated once healing occurs [42]. Antibiotic prophylaxis should be considered from 1 day before until 3 days after the procedure, particularly for patients with prolonged glucocorticoid use and immunosuppression due to MM. Penicillin (with or without a β-lactamase inhibitor) and metronidazole are possible options [42]. To minimize the risk of ONJ and other related complications, a multidisciplinary approach involving oncologists, dentists, and oral surgeons is ideal.

According to the MASCC/ISOO/ASCO guidelines, once a diagnosis of ONJ is established, a staging system should be utilized to determine the extent and severity of the complication to guide clinical management. Currently established staging systems include the 2014 AAOMS staging system, the Common Terminology Criteria for Adverse Events (CTCAE) 5.0, and the 2017 International Task Force on ONJ staging system, with the first two being most frequently applied [73].

The generally accepted treatment objectives for patients with ONJ are pain relief, infection control, and limiting the progression of bone necrosis. Initial conservative measures include analgesics, antimicrobial mouth rinses, systemic antibiotics, and conservative surgical procedures such as the non-invasive removal of superficial sequestered bone. For patients with refractory ONJ and persistent symptoms, more aggressive surgical procedures, such as mucosal flap elevation and block resection, may be considered [73]. Novel treatment approaches currently under research include platelet-rich plasma, hyperbaric oxygen, laser therapy, and parathyroid hormone to establish the efficacy of these treatment methods [74].

## 3. Pain Management from Bone Lesions

### 3.1. Analgesics

The management of MBD requires a multidisciplinary, palliative approach tailored to the disease’s location and whether single or multiple bone metastases are present. Contemporary management has evolved into a multimodal, individualized strategy that incorporates pharmacologic, interventional, and supportive modalities to achieve function-oriented goals for patients.

Traditionally, the pharmacologic treatment of pain in MBD was guided by the World Health Organization’s (WHO) analgesic ladder, developed in 1982 based on limited evidence (Table 1) [75,76]. This approach focused on a step-wise escalation of pain management, starting with non-opioid medications, progressing to weaker opioids, and eventually to stronger opioids [76]. This stepwise approach has been increasingly criticized due to its lack of adaptability to complex cancer pain syndromes like MBD. Currently, the National Institute for Health and Care Excellence (NICE) guidelines are referred to for pain management. These guidelines provide a more comprehensive, multidisciplinary approach that includes non-opioid medications, interventional procedures, and radiotherapy. For mild to moderate pain, paracetamol is recommended due to its renal safety. Whilst NSAIDs were previously used as a first line for short-term relief, their use is now contraindicated in this patient population due to the higher risk of nephrotoxicity [75].

For pain that is not adequately controlled by short-term NSAIDs or weaker opioids (codeine), stronger opioids like morphine, oxycodone, or fentanyl are prescribed. In cases necessitating further pain management, medications such as methadone or buprenorphine can be considered, especially in instances of opioid tolerance [77]. A key aspect of pain management is being mindful of patients’ therapeutic goals, their desired level of functionality, and the impact of pain on their quality of life. Moreover, physicians need to be aware of their cognitive biases to avoid race-based disparities in treating acute or chronic cancer-related pain in minority patients, such as Black patients [78].

For neuropathic components, the NICE guidelines recommend the use of serotonin–norepinephrine reuptake inhibitors (SNRIs), including duloxetine [79]. Particularly, SNRIs can help manage anxiety, depression, and address urinary incontinence, another frequent concern in this patient population [80]. Neuropathic pain can be further managed with anticonvulsants such as pregabalin and gabapentin, alongside topical agents like lidocaine 5% and capsaicin cream, for focal pain relief [81].

### 3.2. Radiotherapy

Radiotherapy (RT) is used as treatment for bone pain in MM in both a palliative and therapeutic care setting, providing pain relief in up to 85% of patients and promoting recalcification in 48% [75]. According to Matuschek et al., the radiosensitive nature of plasma cells makes them vulnerable to RT, leading to the death of cancerous cells, a reduction in nerve compression, and pain improvement [82].

Common indications for RT include the treatment of lytic lesions and spinal cord compression caused by plasmacytomas or vertebral collapse from osteolytic lesions [79]. Spinal cord compression is an oncologic emergency that can be seen in approximately 5–10% of patients with MM throughout their disease [42]. Management includes immediate high-dose corticosteroids to combat edema, MRI imaging, with definitive treatment, including RT or surgical decompression, while accounting for a patient’s clinical status and prognosis [27]. Radiotherapy is highly effective, with up to 90% of patients achieving pain relief. Radiotherapy should be used as an adjunct to surgical fixation for both actual and impending pathological fractures [42]. In these cases, RT aids in bone healing, remineralization, eases pain, reduces the need for pain medication, enhances functional abilities, and decreases the likelihood of future fractures [83]. The RT dose varies according to clinical situations; for example, the palliation of lytic bone lesions may be achieved with 20 to 30 Gy administered in 5 to 10 fractions. Conversely, higher doses of definitive therapy are required to resolve solitary plasmacytomas or to treat spinal cord compression. Despite limited data regarding palliative RT for bone lesions, it is believed that 10 Gy or more will result in partial symptom relief without a dose-dependent response [84].

The use of low-dose RT regimens (4 Gy in 1–2 fractions) was shown to be effective for rapid pain relief and functional improvement, especially in patients with poor marrow reserve or high systemic treatment burden [85]. These regimens offer pain response rates approaching 85–90%, with significantly lower hematologic toxicity and minimal interruption to systemic therapy schedules. Reduction in pain enables increased mobility, restored independence, and lower opioid use, contributing to both physical and mental health improvements. RT also plays a key role in preserving long-term functional independence and QoL [42]. Furthermore, emerging data suggests that RT extends beyond cytotoxicity, influencing the bone microenvironment and promoting a shift from osteolytic to osteoblastic activity, contributing to the stabilization of bone architecture [86]. The combination of RT with immune-modulating therapies, such as immunomodulators or monoclonal antibodies, can enhance local and systemic responses through immunogenic cell death and microenvironmental remodeling [87]. Importantly, newer guidelines now encourage individualized RT planning based on lesion size, pain severity, systemic disease status, and patient frailty, rather than applying a uniform dose–response approach [88].

### 3.3. Interventional Procedures

#### 3.3.1. Vertebroplasty and Kyphoplasty

Regarding skeletal lesions, pathological and impending bone fractures are common in patients with MM. The need for surgical stabilization is up to the discretion of the orthopedic team, but it is typically performed when there is 50% or more cortical bone thickness destruction. Khan et al.’s pooled analysis of twenty-three published case series found that vertebral augmentation (including vertebroplasty and kyphoplasty) resulted in pain reduction immediately after treatment, with the pain relief sustained over time [89]. Table 2 summarizes data from key vertebroplasty and kyphoplasty studies performed in the field of MM. Vertebral fractures may benefit from vertebroplasty, in which polymethyl methacrylate is injected into the affected area, or kyphoplasty, where cement is injected after augmentation with an inflatable balloon [90]. The IMWG recommends both methods for pain relief in MM patients [91]. However, extravasation of cement has been observed in both procedures. Additionally, the extravasation of tumor cells through the vertebral foramina can cause nerve compression in 2–8% of patients, with rare cases of paraplegia reported [92]. Recently, Cornelis et al. used low-pressure stenting to aid in cement deposition and reduce the risk of tumor extravasation. Further validation of this technique is warranted and will require a larger study population and longer follow-up [93].

#### 3.3.2. Surgical Management of Spine Involvement of Myeloma

**Figure 1:**
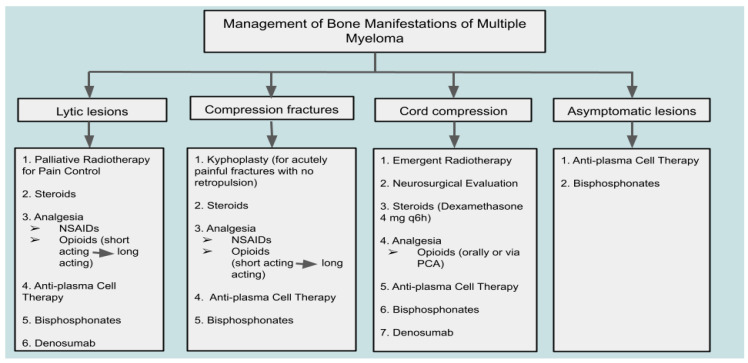
Therapeutic approach to bone disease and complications in multiple myeloma: a clinical management algorithm.

Cord compression in MM patients is typically due to the pathological fracture of the involved vertebral body or the extension of a myeloma lesion within a vertebral body. A rare form of lesion that may cause spinal cord compression is extraosseous myeloma [99]. This epidural lesion should be distinguished from other possible neoplastic lesions, such as lymphomas, meningiomas, and metastatic lesions. While no clear guidelines have been established for spinal cord compression due to myeloma, recommended therapies include radiotherapy and high-dose steroids [99]. According to Rades et al., 76% of MM patients with cord compression who received radiotherapy reported improved motor control, with local control achieved in 98% of patients after one year. Other studies recommend early surgical decompression, followed by radiotherapy or chemotherapy [100]. In an acute setting, presentation of spinal cord compression with urinary or fecal incontinence constitutes a surgical emergency necessitating immediate decompressive intervention to prevent irreversible neurological deficits and improve outcomes [101,102]. Overall, close observation of neurological status and quick identification of deterioration have been widely regarded as key factors in determining emergent versus non-emergent surgical or non-surgical intervention when managing spinal involvement in myeloma [99]. When patients undergo surgical management, they should also be offered palliative care focusing on physical therapy, as well as caregiver education and support to improve overall functional status during the critical post-surgical recovery period [103]. A summary of therapeutic approaches to the management of MBD and skeletal complications in MM is provided in Figure 1.

### 3.4. Novel Therapies (Table 3)

A deeper understanding of the pathogenesis of MBD has helped identify several factors that present promising targets for therapeutic intervention. The following section provides an overview of several novel agents currently undergoing investigation across distinct phases of clinical trials. Positive clinical outcomes from these trials could significantly expand the treatment landscape, offering improved strategies for the prevention and management of MBD.

**Table 3:** Trials for novel therapies for myeloma bone disease.

Drug Name	Trial Results	Key Effectiveness Data	Reported Drug-Related Adverse Events (Incidence %)
Sotatercept (ACE-011)	Phase I: Increased bone formation and decreased bone resorption in post-menopausal women	Increase in BSALP **: 3.0 mg/kg IV group: Peak increase of 35.9% from baseline at day 15 Placebo group: No significant changes in BSALP at any time point Change in CTX ^‡^ from baseline at day 15: 3.0 mg/kg IV group: 24.1% decrease Placebo group: 2.6% increase	
	Phase IIa: Improved bone formation markers, increased hemoglobin levels, reduced bone pain	Improvement in bone pain (VAS ^¶^ score): Mean decrease of 5.8 to 12.7 (sotatercept) vs. −0.7 to 7.2 (placebo)	Hypertension (12.5)
BHQ880	Phase I/II: BHQ880 with zoledronic acid increased spine and hip bone strength	Increase in bone strength at the hip: 2.4% to 3.7% (baseline to EOT ^†^) Increase in bone strength at the spine: 2.8% to 20.2% (baseline to EOT ^†^)	Hypertension (7.1) Raised creatinine (3.6) Thrombocytopenia (3.6)
	Phase II: Increased bone anabolic activity in high-risk smoldering MM	Anabolic bone activity: Vertebral strength increase: 3% from baseline (*p* = 0.002), exceeding 5% in some cases	Arthralgia (16) Fatigue (12) Pain in extremity (12) Pyrexia (12)
Romosozumab	Phase III: Decreased vertebral fracture risk in postmenopausal women with osteoporosis	Reduction in new vertebral fractures: 73% lower risk (vs. placebo; RR = 0.27, *p* < 0.001)	Arthralgia (13) Nasopharyngitis (12.8) Back pain (10.5)
	Preclinical: Bone loss prevention, increased bone strength		
Tanezumab	Phase III: Improved average daily pain	≥50% improvement in average pain: 25.4% (vs. 12.3% placebo; OR = 2.55, *p* = 0.0405)	Anemia (8.3) Arthralgia (8.3) Decreased appetite (8.3)

** BSALP: Bone-specific alkaline phosphatase, ^‡^ CTX: C-terminal type 1 collagen telopeptide, ^¶^ VAS: Visual Analog Scale, ^†^ EOT: End of Treatment.

#### 3.4.1. Activin Inhibitors

Activin, a member of the TGF-β superfamily, plays a critical role in bone remodeling by acting as an osteoclast agonist and osteoblast antagonist, thereby contributing to the pathogenesis of MBD. Elevated levels of activin-A have been observed in MM patients and are thought to be correlated with advanced disease, extensive bone involvement, and poorer survival outcomes [104]. Accordingly, activin is an intriguing target for drug development.

Sotatercept (ACE-011), a fusion protein comprising the extracellular domain of the high-affinity activin receptor type IIA (ActRIIA) and the human immunoglobulin G (IgG) Fc domain, binds to activin A/B and other members of the TGF-β superfamily and can disrupt downstream signaling pathways. Consequently, sotatercept exerts potent inhibitory effects on osteoclast function of bone resorption [105]. In a murine model of MM, RAP-011, the murine counterpart of sotatercept, effectively restored osteoblast function and inhibited MM-induced osteolysis [106]. In a Phase I study involving healthy postmenopausal women, sotatercept increased bone formation markers, such as bone-specific alkaline phosphatase (bALP), and decreased bone resorption markers (CTX and TRACP-5b), indicating a favorable effect on bone turnover [107]. Additionally, a multicenter Phase II trial randomized MM patients with osteolytic lesions to receive either four 28-day cycles of sotatercept or placebo as a subcutaneous injection with concomitant anticancer therapy consisting of oral melphalan, prednisolone, and thalidomide. Those treated with sotatercept had clinically significant increases in bone formation biomarkers, reduced bone pain, increased hemoglobin levels, and demonstrated antitumor activity [108]. Furthermore, ongoing clinical trials, such as NCT01562405, are exploring sotatercept in combination with lenalidomide/dexamethasone or pomalidomide/dexamethasone, for the reduction or prevention of cancer cell growth, as well as to improve anemia and bone lesions in MM patients [109].

Similarly, luspatercept is an erythroid maturation agent that functions as a ligand trap for activins [110]. While luspatercept has demonstrated clinical efficacy in treating anemia, its role in modifying bone turnover in MM remains unclear. In the Phase III BELIEVE trial, patients with transfusion-dependent β-thalassemia treated with luspatercept maintained stable bone health over 96–192 weeks, with no statistically significant changes in bone mineral density at the hip or lumbar spine compared to baseline [111]. Preclinical murine studies indicate that luspatercept stimulates osteoblast maturation and mitigates bone loss in models of myelodysplastic syndrome, suggesting potential bone anabolic effects, but there are no studies specific to MM [112].

#### 3.4.2. DKK-1 Antagonists

DKK-1, an endogenous Wnt pathway inhibitor, plays a crucial role in the dysfunction of osteoblasts observed in MM. Produced by MM cells, DKK-1 inhibits osteoblast maturation and new bone formation, leading to the development of osteolytic lesions. High levels of serum DKK-1 are associated with advanced disease features and extensive bone involvement in MM patients. Elevated DKK-1 levels have been observed in symptomatic MM patients at diagnosis and relapse, correlating with the presence of lytic lesions, while levels in asymptomatic patients are similar to control values [113].

BHQ880, a fully human monoclonal antibody that neutralizes DKK-1, has shown promise as a therapeutic agent for MM-related bone disease. Preclinical studies using murine models of myeloma have demonstrated that inhibiting DKK-1 with BHQ880 promotes bone formation, reduces the development of osteolytic lesions, and inhibits MM cell proliferation by altering the bone marrow microenvironment. These effects are attributed to osteoblast function restoration, osteoclastogenesis reduction, and increased bone mineral density in myeloma-affected bones [114]. Clinical trials have further explored the potential of BHQ880 in treating MM. In a Phase IB study, BHQ880 combined with zoledronic acid showed increased bone strength at the spine and hip in relapsed or refractory MM patients with a prior SRE [115]. A Phase II open-label study (NCT01302886) evaluating BHQ880 in high-risk smoldering MM demonstrated increased bone anabolic activity, although its antitumor effects are still under investigation [116]. Overall, targeting DKK-1 represents a promising therapeutic strategy for mitigating bone disease and potentially inhibiting tumor growth through modifications of the bone marrow microenvironment.

#### 3.4.3. Sclerostin Antagonists

Sclerostin, a cysteine knot-containing protein produced by osteocytes, is a critical regulator of bone metabolism, particularly in bone remodeling [113]. It functions by inhibiting osteoblast-driven bone formation and inducing apoptosis in mature osteoblasts. Sclerostin achieves this by inhibiting the Wnt/β-catenin signaling pathway, a key intracellular cascade involved in promoting bone formation. Sclerostin deficiency leads to rare bone sclerosing disorders, while elevated levels of sclerostin are implicated in the pathogenesis of metabolic bone diseases like postmenopausal osteoporosis [117]. Additionally, high circulating levels of sclerostin are associated with abnormal bone remodeling and increased fracture risk in patients with symptomatic MM. MM patients who presented with fractures at diagnosis were found to have very high levels of circulating sclerostin compared with all others (*p* < 0.01), while sclerostin serum levels correlated negatively with bALP (r = −0.541; *p* < 0.0001) and positively with CTX (r = 0.524; *p* < 0.0001) [105].

Romosozumab, a humanized monoclonal antibody that neutralizes sclerostin, has shown significant potential in treating bone-related disorders. Clinical trials in postmenopausal women with osteoporosis have demonstrated that romosozumab effectively increases bone mineral density and reduces the risk of vertebral fractures [118]. In preclinical studies, romosozumab has been effective in preventing bone loss whilst enhancing bone strength in models of MBD, potentially offering superior benefits compared to ZA [119]. Furthermore, preclinical studies testing the combination of sclerostin antibodies with anti-MM drugs, such as bortezomib and dexamethasone, found that these antibodies did not affect or interfere with the anti-MM activity of drugs. Accordingly, sclerostin antibodies and anti-MM drug combination therapy may be beneficial in improving both bone disease and inhibiting tumor progression [120].

#### 3.4.4. Nerve Growth Factor (NGF) Inhibitors

NGF plays a key role in pain modulation by binding to the tropomyosin receptor kinase A, facilitating the transmission of pain signals through the regulation of ion channels. Elevated levels of NGF have been observed in chronic pain conditions, making it a promising therapeutic target [75]. Tanezumab, a monoclonal antibody against NGF, has been found to improve pain and function in chronic pain conditions such as osteoarthritis and chronic low-back pain [121]. A recent phase III trial (NCT02609828) assessed the efficacy and safety of subcutaneous tanezumab in subjects with pain due to cancer metastasized to bone or multiple myeloma. Participants were randomized to receive either a placebo or 20 mg of tanezumab. In this study, tanezumab met the primary endpoint by demonstrating greater improvement in daily average pain intensity at the index bone metastasis cancer pain site at week 8 compared to the placebo, highlighting the potential of anti-NGF therapies to reduce cancer pain [121].

### 3.5. Quality of Life and Patient Considerations

Management of MBD must go beyond preventing SREs to improve the quality of life for patients. Bone pain, limited mobility, fatigue, and fear of fractures can significantly affect patients’ physical function and emotional well-being. Bone disease contributes to reduced functioning, loss of independence, and psychosocial distress, all of which negatively impact QoL scores on validated tools, including EORTC QLQ-C30 and the MY20 modules [122,123]. Therapeutic decisions should incorporate the patient’s functional goals, risk tolerance, and values, especially when weighing continued antiresorptive therapy or surgical interventions [124]. Additionally, the burden of frequent hospital visits for intravenous bisphosphonates or imaging, along with concerns about long-term complications such as ONJ or renal toxicity, must be acknowledged. Integrating early palliative care, physiotherapy, and psychological support into standard bone disease management can improve both symptom burden and patient satisfaction [125,126]. Numerous patient factors should be considered, including renal function, fracture risk, disease burden, performance status, co-morbidities, and crucially, psychosocial factors. Therefore, individualized care plans that prioritize pain control, functional preservation, and shared decision-making, accounting for the patient’s preferences, are essential to maintaining quality of life in patients with MBD.

### 3.6. Biomarkers

Despite advances in therapeutics, heterogeneous bone involvement and variable responses to treatment continue to challenge the management of MBD. Emerging research emphasizes a shift toward biomarker-driven strategies that leverage molecular, biochemical, and imaging indicators to personalize patient care, improve risk stratification, and optimize therapeutic efficacy.

Recent data reinforces the dynamic role of Wnt signaling inhibitors, particularly DKK-1 and sclerostin, as actionable biomarkers in MM [16,17]. A study of newly diagnosed MM patients used serial measurements of serum DKK-1 and sclerostin to correlate with disease stage and bone lesion burden, showing marked declines in these markers after effective therapy, thus reinforcing their utility in monitoring disease activity and therapeutic response [120,127]. Furthermore, the 2024 Nature Reviews Disease Primers update underscores the microenvironmental interactions and signals like Wnt and RANKL that disrupt bone homeostasis, highlighting opportunities for targeted biologic interventions and personalized monitoring [128].

Moreover, future trials could integrate biomarker thresholds, including elevated DKK-1 or sclerostin, to tailor the initiation, dosing, or tapering of bone-targeted agents. The ongoing development of composite biomarker panels like Wnt antagonists, bone turnover markers (CTX/PINP), and bone marrow cytokines (activin A, IL-6), coupled with advanced imaging modalities such as whole-body DW-MRI or PET/CT, offers an avenue toward risk-adapted care. Early-phase trials using biomarker-guided therapy decisions are essential to validate this precision framework and pave the way toward more adaptive, biologically informed treatment algorithms for MBD.

## 4. Conclusions

MM is a chronic, progressive hematologic malignancy that requires a comprehensive treatment paradigm integrating both disease-modifying therapies and supportive care measures. Among the most debilitating complications of MM is MBD, which significantly impairs functional status and quality of life. Effective management of MBD necessitates a multidisciplinary, patient-centered approach, involving hematologists, radiation and orthopedic oncologists, palliative care specialists, and active patient engagement. Aligning treatment with individual patient goals is essential for optimizing outcomes and preserving quality of life.

The therapeutic landscape of MM has advanced with the advent of proteasome inhibitors, immunomodulatory drugs, monoclonal antibodies, histone deacetylase inhibitors, selective inhibitors of nuclear export, NK cell–based therapies, and anti-BCMA immunotherapies. These innovations have substantially improved disease control and survival. However, the burden of skeletal complications persists, underscoring the need to elevate supportive care to a central component of myeloma management. Recent advances in the understanding of bone biology, the tumor microenvironment, and the pathophysiology of MBD have catalyzed the development of novel bone-targeted agents.

Therefore, as myeloma-directed therapies evolve, equal emphasis must be placed on advancing supportive strategies for bone disease. Future research should prioritize the integration of novel bone-targeted agents into MM treatment algorithms, supported by clinical trials and biomarker-driven approaches. This dual focus on prolonging survival and enhancing quality of life will be essential for delivering truly comprehensive care in multiple myeloma.

## Figures and Tables

**Table 1:** Bone therapies: existing and novel drugs, classes, modes of action, therapeutic targets, and effects on bone.

Drug	Mode of Action	Therapeutic Target	Effect on Bone
Bisphosphonates	Pyrophosphate analog	Osteoclast inhibition via inhibition of the mevalonate pathway	Reduces bone resorption and turnover, stabilizes bone lesions, prevents skeletal-related events (SREs)
Denosumab	Human monoclonal antibody (IgG2)	RANKL so inhibits osteoclast formation	Inhibits bone resorption and stabilizes lytic lesions
Sotatercept (ACE-011)	Activin A neutralizing receptor, a recombinant activin receptor type IIA (ActRIIA) ligand trap	Osteoclast inhibition and osteoblast maturation	Increases bone mineral density and
BHQ880	DKK-1 neutralizing monoclonal antibody	Osteoblast maturation and function restoration through Wnt pathway inhibition	Increases bone mineral density and trabecular volume
Romosozumab	Sclerostin-neutralizing monoclonal antibody	Increased osteoblast differentiation and activity Decreased osteoclastogenesis	Reverses lytic lesions and promotes bone remineralization
Tanezumab	Monoclonal antibody against Nerve Growth Factors (NGFs)	Prevents NGF from interacting with TRk receptors to block signaling pathways	No direct effects, changes in pain perception, and mechanical load

**Table 2:** Summary of key vertebroplasty and kyphoplasty studies in multiple myeloma.

Study Title, Year	Study Design, *N*	Intervention	Key Findings
Percutaneous balloon kyphoplasty to treat pathological vertebral body fracture and deformity in MM: a one-year follow-up, 2006 [94]	Prospective cohort, 20	Balloon kyphoplasty	1. Pain reduction: Significant decrease in VAS * (*p* < 0.05) 2. Oswestry improvement: Significant reduction (*p* < 0.05) 3. Vertebral height: Stabilized postoperatively 4. Complications: 10.4% asymptomatic cement leakage
Vertebroplasty in MM: a Large Patient Series, 2008 [95]	Retrospective Review, 67	Vertebroplasty	1. Pain reduction: Rest pain ↓2.7 points (*p* < 0.001); Activity pain ↓5.3 points (*p* < 0.0001) 2. Seventy % improved mobility 3. RDQ ** improvement: 11.0 points (*p* < 0.0001)
Percutaneous vertebroplasty in MM, 2011 [96]	Prospective cohort,106	Vertebroplasty	1. Pain reduction: VAS decreased from 9 to 1 2. ODI improvement: Decreased from 82% to 7% 3. Brace use: 86% discontinued the brace All outcomes had *p* < 0.001
Safety and efficacy of percutaneous vertebroplasty in malignancy, 2011 [97]	Systematic Review, 987	Vertebroplasty	1. Pain reduction: 47–87% 2. Complications: (5 deaths, 19 major adverse events)
Vertebral Augmentation in Patients with MM, 2014 [89]	Systematic Review, 923	Vertebral augmentation (kyphoplasty/vertebroplasty)	1. Pain reduction: -4.4 points at ≥1 year (*p* < 0.001) -No difference between vertebroplasty and kyphoplasty (*p* > 0.9) 2. ODI ^‡^: No significant improvement (*p* > 0.05) 3. Analgesic use: Significant reduction at 1 year (85%, *p* = 0.003)
Comparison if the addition of multilevel vertebral augmentation to conventional therapy will improve the outcome of patients with MM, 2016 [98]	Prospective cohort, 27	Group I: Conventional Group II: Conventional + Vertebroplasty + Kyphoplasty	Group II (augmentation): 1. Improved ODI (*p* = 0.047) and SINS ^¶^ (*p* = 0.002) vs. Group I 2. Mortality: Equal in both groups (4 deaths each)

* VAS: Visual Analog Scale, ** Roland Morris Disability Questionnaire, ^‡^ Oswestry Disability Index, ^¶^ Spinal Instability Neoplastic Score.

## Abbreviations

The following abbreviations are used in this manuscript:

MM	Multiple Myeloma
MBD	Myeloma bone disease
ZA	Zoledronic Acid
ONJ	Osteonecrosis of the Jaw
VGPR	Very good partial response
OPG	Osteoprotegerin
RT	Radiotherapy
NGF	Nerve Growth Factor
SNRIs	Serotonin-norepinephrine reuptake inhibitors
SMM	Smoldering multiple my
MGUS	monoclonal gammopathy of undetermined significance
OS	Overall survival
PFS	Progression-free survival
ASCO	American Society of Clinical Oncology
IMWG	International Myeloma Working Group
NCCN	National Comprehensive Cancer Network
